# Adherence of the #Here4U App – Military Version to Criteria for the Development of Rigorous Mental Health Apps

**DOI:** 10.2196/18890

**Published:** 2020-06-17

**Authors:** Brooke Linden, Linna Tam-Seto, Heather Stuart

**Affiliations:** 1 Health Services and Policy Research Institute Queen's University Kingston, ON Canada

**Keywords:** mental health services, telemedicine, mHealth, chatbot, e-solutions, Canadian Armed Forces, military health, mobile phone

## Abstract

**Background:**

Over the past several years, the emergence of mobile mental health apps has increased as a potential solution for populations who may face logistical and social barriers to traditional service delivery, including individuals connected to the military.

**Objective:**

The goal of the #Here4U App – Military Version is to provide evidence-informed mental health support to members of Canada’s military community, leveraging artificial intelligence in the form of IBM Canada’s Watson Assistant to carry on unique text-based conversations with users, identify presenting mental health concerns, and refer users to self-help resources or recommend professional health care where appropriate.

**Methods:**

As the availability and use of mental health apps has increased, so too has the list of recommendations and guidelines for efficacious development. We describe the development and testing conducted between 2018 and 2020 and assess the quality of the #Here4U App against 16 criteria for rigorous mental health app development, as identified by Bakker and colleagues in 2016.

**Results:**

The #Here4U App – Military Version met the majority of Bakker and colleagues’ criteria, with those unmet considered not applicable to this particular product or out of scope for research conducted to date. Notably, a formal evaluation of the efficacy of the app is a major priority moving forward.

**Conclusions:**

The #Here4U App – Military Version is a promising new mental health e-solution for members of the Canadian Armed Forces community, filling many of the gaps left by traditional service delivery.

## Introduction

### Background

Recent advances in and increased access to technology have created opportunities to shift mental health support services away from traditional person-delivered models toward those that are more technologically based, including those that may be provided through smartphone apps. The ease of use and availability of these apps are ideal for populations who may face logistical or social barriers to traditional service delivery, including members of the Canadian Armed Forces (CAF).

Military life is characterized by frequent relocations and family absences, and increased risk for illness, injury, and death [1]. Research has also identified how military life can affect health and well-being, including increasing risk for mental health difficulties and challenges with accessing health care services [2-4]. These events have been shown to impact all members of the community, including personnel [3,5,6], veterans [7], and their family members [8-10]. Despite the prevalence of mental health challenges faced by members of the CAF community, traditional mental health care continues to be underused. Commonly cited barriers include a preference for self-management [11], fear of impact on one’s military career [11], perceived stigma [12], and structural barriers such as living in remote locations and being frequently relocated [5]. As a result, a technology-based solution may better serve members of the military community.

**Figure 1:**
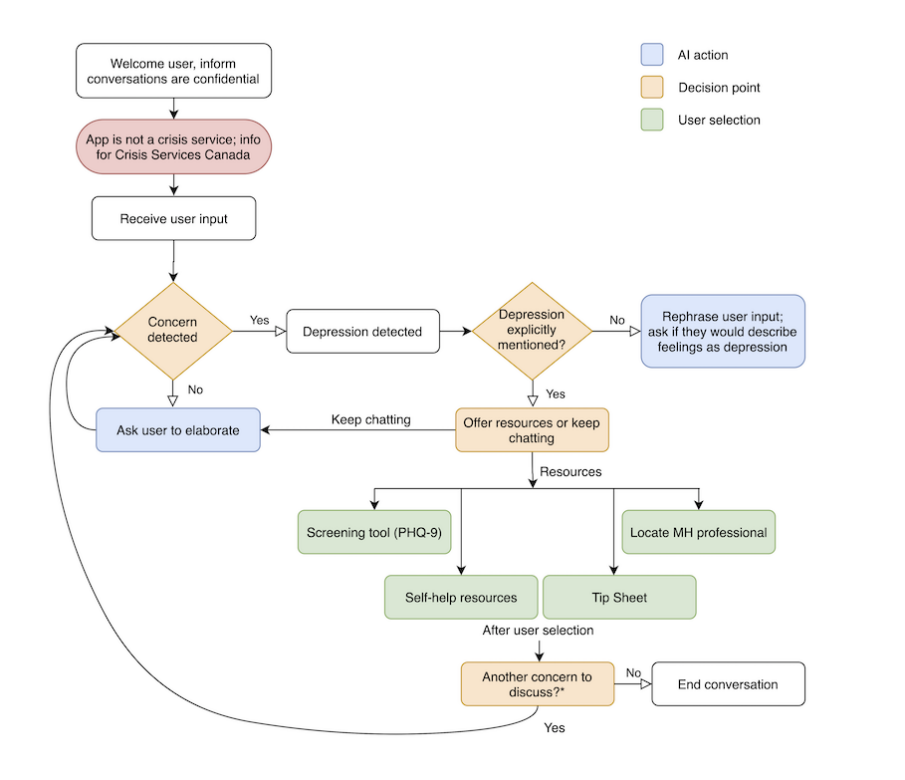
Demonstration of the basic functionality of the Here4U app, using depression as an example of a pressing concern. If the user has indicated an additional concern, the subsequent conversational flow (and end point options or resources provided) would change to reflect the new concern. *An additional concern may be expressed by the user at this point (AI prompt: “Is there anything else you wanted to chat about today?”) or from appended additional concerns (eg, if more than one concern is detected at the beginning of the conversation). AI: artificial intelligence; MH: mental health; PHQ-9: Patient Health Questionnaire-9.

The #Here4U App – Military Version (hereafter, #Here4U) is an app designed to provide evidence-informed mental health support to members of the CAF community, including serving personnel, veterans, and their adult family members. #Here4U uses artificial intelligence (AI) in the form of IBM’s Watson Assistant to carry on unique text-based conversations with users, identify presenting mental health concerns, and refer users to self-help resources or recommend professional health care where appropriate. Users are invited to converse with the chatbot and select their end point (ie, self-help solution) from several options, facilitating a user-directed experience. Options include (1) tip sheets, which provide the user with brief mental health education on the selected topic; (2) use of validated screening tools, which provide the user with a self-assessment of the severity of psychological distress they may be experiencing; (3) information on how to contact local supports, including counsellors, psychologists, and psychiatrists; (4) frequently asked questions (FAQs) about common mental disorders, offered as downloadable PDF; and (5) self-help resources, ranging from cognitive behavioral therapy (CBT) education and activities to simple at-home exercises such as tactical breathing, meditation, and progressive muscle relaxation. The beta version of #Here4U has been designed to serve members of the CAF, veterans, and adult military family members, with plans to expand the app’s applicability to additional target groups in the future. Figure 1 demonstrates the functionality of the app, using a flowchart design.

The #Here4U app was developed as an interdisciplinary project, in partnership with IBM Canada, the Queen’s University Centre for Advanced Computing, Kingston, ON, Canada, and the Queen’s University Health Services and Policy Research Institute. In addition to the research team, subject matter experts were engaged throughout the development process to share their experiences regarding common presenting mental health concerns among members of the CAF community, review resources provided by the app, and provide professional input and guidance on conversational flows. Subject matter experts included social workers, physicians (specializing in family medicine and psychiatry), psychologists, first responders, military family health researchers, child and youth workers, and members of the CAF community (active members, military spouses, family of adult military members, and veterans).

### Objective

As the availability and use of mental health apps has increased, so too has the list of recommendations for efficacious development. In 2016, Bakker and colleagues published a set of 16 evidence-based recommendations to support the development of rigorous apps [13]. Our objective is to demonstrate the rigorous development and testing processes undertaken between 2018 and 2020 to produce the beta version of the #Here4U app and, in doing so, evaluate the degree to which the app complies with each of the criteria laid out by Bakker and colleagues.

## Methods

### Development of the App

The first year of development included background research to inform the content of the app, in addition to the initial development of conversational flows. First, we undertook a preliminary study to determine the most common presenting mental health challenges within the CAF community, as well as to derive particular language and slang used in the community to discuss mental health–related challenges. This was necessary to train IBM’s Watson Assistant (IBM Corporation) to be able to recognize and interpret specific vernacular used within the community. We conducted a combination of online surveys (n=12), focus groups (n=17), and individual interviews (n=14) to achieve the goals of this phase of development, the results of which are described in detail elsewhere (available from the authors upon request). Ultimately, we identified 3 presenting concerns as the primary areas of focus: generalized anxiety, depression, and posttraumatic stress disorder (PTSD). We also identified several secondary concerns as priorities for later development (eg, substance use and addictive behaviors, general stress, difficulties related to sleep).

Next, we drew up initial logic trees to map likely conversational flows. These were then coded into the back end of the AI by members of the computing team. We initially designed conversational flows for secondary concerns to be more streamlined (ie, to program the AI to recognize the concern expressed and immediately offer potential resources), whereas flows for primary concerns were more intricate. From here, we selected useful, efficacious end points for these conversations with input from subject matter experts (eg, validated screening tools, evidence-based self-help resources and activity recommendations, tip sheets to facilitate improved mental health education).

### Beta Testing

During the second year of development, we used a snowball sampling method to gather participants to test the beta version of the app. Participants were a mix of subject matter experts involved in app development, attendees at the Canadian Institute for Military and Veteran Health Research Forum in 2019, members of the CAF community (eg, active members, veterans, military family members, staff at Military Family Resource Centres, mental health professionals working within the military community), and personal contacts of members of the research team who were connected to military research in some way (n=93). Users were asked to engage in conversations with the app, submit their comments regarding usability and suggestions for improvement, and identify any problems with the conversation dialogue. For example, testers identified instances in which the AI misunderstood a term or comment, or drew incorrect conclusions, such as placing the user into a conversational flow for anxiety when they were expressing concerns related to depression. This allowed us to refine and expand the AI’s vocabulary, as well as to observe areas in need of improvement or expansion in the app. We made changes to the app in response to tester feedback using a waterfall approach, whereby we corrected conversational flows after receiving feedback from each tester.

### Assessment of Rigorous Development

Finally, prior to conducting a formal evaluation of the app, we sought to assess whether we had met expectations regarding the recommendations for rigorous app development. We referred to Bakker and colleagues’ 16 criteria, outlining the degree to which the #Here4U app met (or did not meet) each of these criteria, emphasizing relevant features and functions of the app in doing so.

### Ethics

All stages of research conducted to support the development of the #Here4U app were approved by the Queen’s University Health Sciences and Affiliated Teaching Hospitals Research Ethics Board. The research project was also approved by the Canadian Department of National Defence/CAF Social Science Research Review Board in accordance with Defense Administrative Orders & Directives 5062-0 and 5062-1 (#1172/18F).

## Results

In this section, we address each of the 16 criteria put forward by Bakker and colleagues, highlighting where the #Here4U app met expectations and where selected criteria were not applicable.

### Recommendation 1: Cognitive Behavioral Therapy Based

CBT is a common therapeutic approach used to treat several mental illnesses, such as anxiety and depression, designed to help individuals develop more adaptive methods of thinking and processing emotions and behavior [14]. The #Here4U app met this recommended criterion by providing users with CBT-based self-help resources, in the form of exercises and self-help workbooks. Users are also invited to access psychoeducational resources regarding the purpose and principles of CBT, including what the cognitive behavioral model looks like, and how to challenge problematic thinking by using tools such as recognizing cognitive distortions, decatastrophizing, and reframing.

### Recommendation 2: Address Both Anxiety and Low Mood

**Figure 2:**
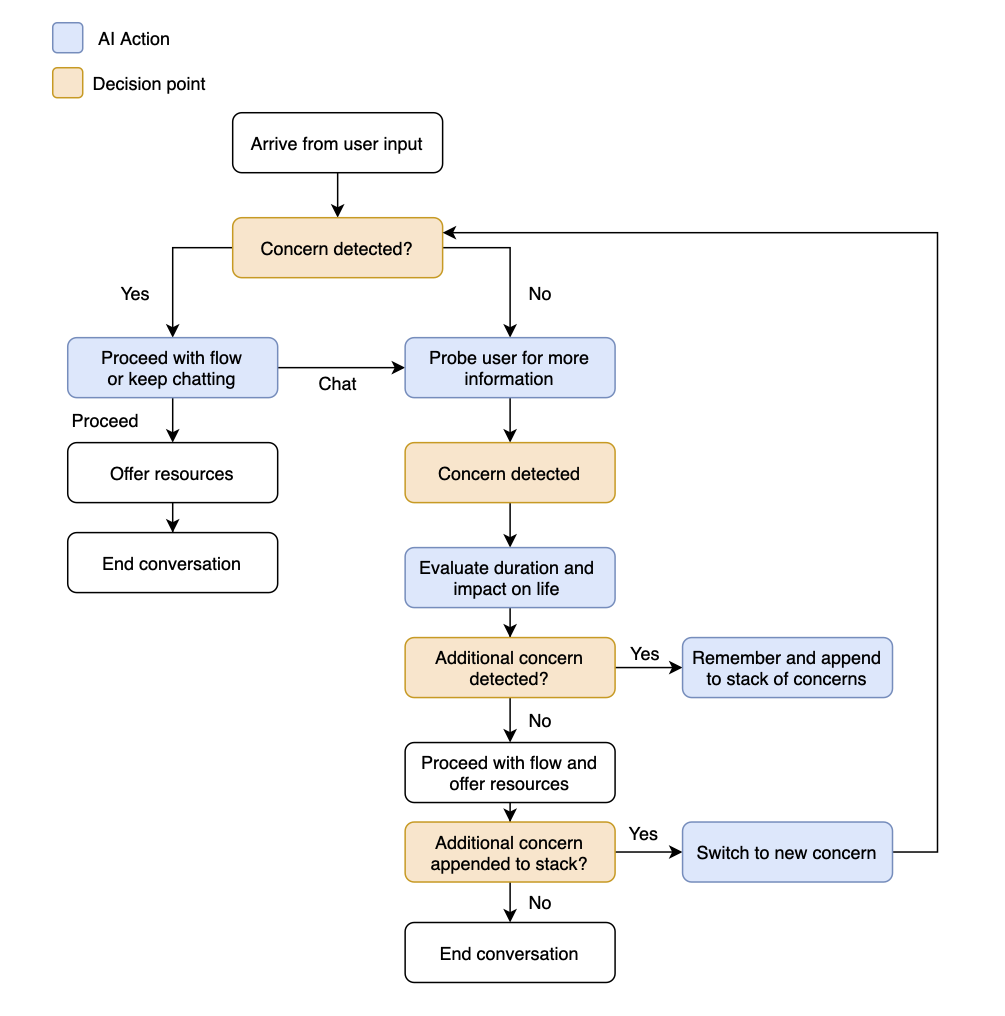
Demonstration of the loop-back function in the Here4U app. AI: artificial intelligence.

The #Here4U app met this criterion by addressing 3 major mental health concerns common among the CAF community: anxiety, depression, and PTSD. We selected these concerns after primary research conducted among members of the CAF community revealed these to be the most common mental health concerns. User-led conversational flows and self-help resources are provided for each of these areas of concern, in addition to validated screening measures for assessing degree of distress [15-17]. The app is also equipped to manage multiple presenting concerns at a given time, using a “loop-back” function. For example, anxiety, depression, and PTSD are often comorbid conditions, among both the general population [18] and the military population [19]. If a user were to express concerns associated with more than one mental health concern in a single conversation, the Here4U app would initially direct the conversation toward the first concern, later returning—or looping back—to discuss additional challenges. In the event that a user expresses both primary and secondary concerns, the app was designed to prioritize primary concerns (eg, depression, anxiety, and PTSD), prior to returning to address secondary concerns. Figure 2 depicts the loop-back function. The ability to address multiple concerns during a single conversation ensures that users receive the most complete mental health information, in addition to eliminating the need to juggle multiple apps or multiple sessions to address different concerns.

### Recommendation 3: Designed for Use by Nonclinical Populations

The #Here4U app was specifically designed for use among nonclinical populations (ie, individuals who do not have a clinical diagnosis for a mental health disorder), with the app intended to be broadly applicable to a variety of individuals within the CAF community. While some of the screening tools contained in the app can be used to determine a clinical diagnosis when administered by a mental health professional (eg, the Patient Health Questionnaire-9), they are explicitly used here as measures of severity [16,17]. Apps that provide users with a likelihood of a clinical diagnosis have the potential to be harmful where users may be tempted to self-diagnose a mental illness when they may simply be experiencing low levels of distress [20]. Rather than providing a diagnosis or treatment, the #Here4U app was intended as an upstream measure (eg, with focus laid on mental health promotion and mental illness prevention). While the app can assist with directing users who may be experiencing more serious levels of psychological distress to a mental health professional for additional enquiry, the resources offered within the app were designed to provide users with the education to self-manage nonclinical, low levels of distress.

### Recommendation 4: Automated Tailoring

The #Here4U app achieved automated tailoring through the use of IBM’s Watson Assistant AI agent, which is capable of interpreting user concerns (eg, referred to technically as entities, or key words, and intents, or key phrases) and connecting users to the most appropriate conversational flow. With this function, users do not have to sort through menu items to locate the information that is most relevant or useful to them.

As previously noted, the #Here4U app was designed to respond to 3 primary mental health concerns (anxiety, depression, and PTSD), each with its own end point solutions offered, rather than using a one-size-fits-all approach. Secondary conversational flows within the app allow for even more detailed tailoring. For example, changes in appetite and sleep are commonly linked to depression and anxiety among members of the CAF community [21,22], while experiencing chronic stress or pain can similarly lead to mental health difficulties [23,24]. Additionally, substance use and addictive behaviors are often adopted as negative coping mechanisms [25-27]. Many members of the CAF community have reported experiencing family-related challenges, such as frequent absences, child rearing, supporting an injured family member, and frequent relocations. In addition to primary concerns, the #Here4U app also offers brief conversational flows that provide resources for users experiencing any of these secondary concerns.

### Recommendation 5: Reporting of Thoughts, Feelings, or Behaviors

While the #Here4U app does not track or store user information (see recommendation 12), this criterion was met by providing users with self-help resources that encourage the reporting of thoughts, emotions, and behaviors to improve awareness of changes in one’s mental health. For example, one option for users expressing concerns related to low mood is to begin using a daily gratitude journal, where users are invited to record three “good things” that happen every day. Users expressing challenges associated with stress management are provided with step-by-step guidelines on how to use progressive muscle relaxation as a stress reduction technique. All self-help resources provided through the app have been extracted or adapted from established programs (eg, Anxiety Canada) and have been reviewed by mental health professionals.

### Recommendation 6: Recommend Activities

Many activities offered through the #Here4U app focus on mood improvement, such as encouraging users to engage in hobbies, leisure time, and physical activity and to spend time in nature. Behavioral activation is encouraged through CBT-based activities, including goal setting, cognitive distortion reframing, and decatastrophizing. Finally, users are invited to develop coping skills through improving their understanding of the importance of having a strong social support system and the adoption of coping activities such as progressive muscle relaxation, meditation, and tactical breathing.

### Recommendation 7: Mental Health Information

The #Here4U app met this criterion by providing users with psychoeducation by offering tip sheets for anxiety, depression, and PTSD, as well as more detailed FAQ documents that cover areas such as common symptoms, treatments, and how to help someone else who may be struggling. General education on CBT techniques for improving and managing mental health is also provided as an option for users. All materials were developed using evidence-based, reputable resources and have been reviewed by mental health professionals to ensure accuracy of information.

### Recommendation 8: Real-Time Engagement

**Figure 3:**
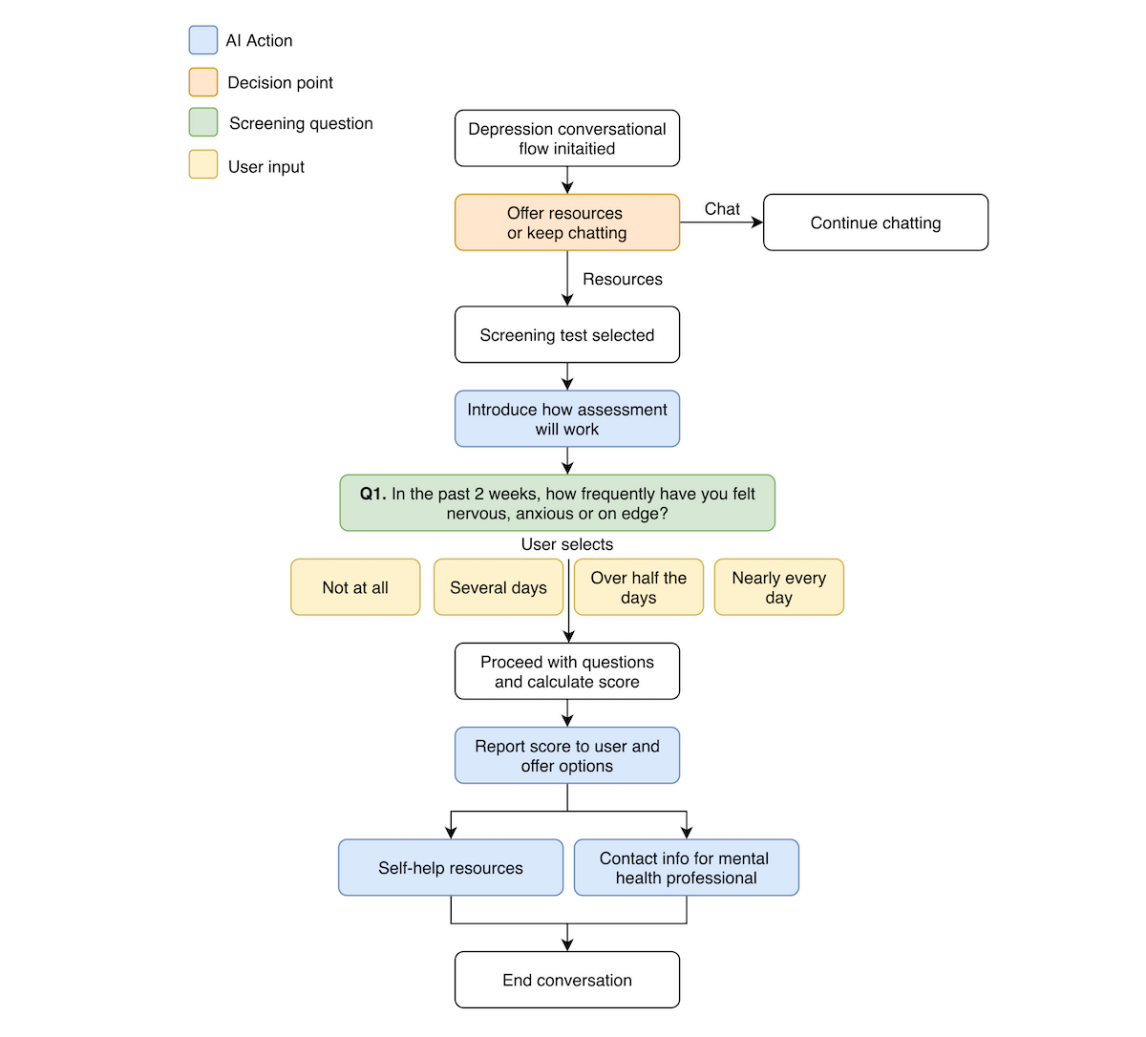
Demonstration of the functionality of the question-and-answer screening test in the Here4U app. AI: artificial intelligence.

The #Here4U app capitalizes on the real-time capabilities of mobile devices by encouraging users to engage with the recommended self-help activities and resources immediately upon accessing them. Within the app, users can complete screening tests, which provides them with an idea of the degree to which they may be experiencing psychological distress, using an interactive, question-and-answer format (Figure 3). While these screening tests do not provide users with a diagnosis due to the risks outlined in recommendation 3, the app can also link users to the *Psychology Today* search engine to help them find a psychologist or counsellor in their local area [28] or to the Calian Military Family Health Portal [29], where users can get help finding a family physician. Finally, the 24/7 availability of the app encourages users to reach out and access resources in real time whenever they experience a change in their mental health.

### Recommendation 9: Activities Explicitly Linked to Specific Reported Mood Problems

With its tailoring capabilities, combined with the ability to address multiple presenting issues in a single session, the #Here4U app links reported mood challenges to relevant activities, rather than using a one-size-fits-all approach. While there is some overlap between concerns (eg, CBT educational materials are provided for all users), a user experiencing symptoms of depression would be directed toward activities and self-help resources specifically designed to address low mood. Bakker and colleagues argued that linking activities in this way can improve engagement and encourage habit-forming use of the app [13].

### Recommendation 10: Encourage Nontechnology-Based Activities

The #Here4U app met this criterion by encouraging users to engage in a selection of nontechnology-based activities to improve their mental health (eg, engaging in regular physical activity, healthy nutrition, or self-care hobbies or spending time in nature). All of these recommended activities have been linked to reducing stress and improving low mood and anxiety [30-33].

### Recommendation 11: Gamification and Intrinsic Motivation to Engage

Gamification, or the use of game-based mechanics to improve users’ engagement, learning, and problem solving, can help to counteract problems related to lack of motivation and improve goal setting [13]. Currently, the #Here4U app does not contain any elements of gamification, but this may be an area for future development. Based on data derived from focus groups and interviews, gamification is a feature that potential users may find beneficial with respect to encouraging ongoing engagement with the app.

### Recommendation 12: Log of Past App Use

During the first phase of research for the project, we consulted members of the CAF community and subject matter experts (eg, psychologists, counsellors, social workers) to determine, among other things, common barriers to using a mental health app. The most commonly cited barriers were the need for privacy and confidentiality, most often driven by concerns regarding the potential for mental illness stigma resulting in damage to one’s military reputation or career. For military families using the app, there was the concern that any kind of mental health challenge or family difficulty would reflect poorly on the military member. As a result of these findings, we made an informed decision to not implement any feature that would track app history. Bakker and colleagues mainly referred to this recommendation as a component of gamification, where users can track their progress week over week [13]. As the #Here4U app does not contain elements of gamification, logging past app use in this manner was not applicable.

### Recommendation 13: Reminders to Engage

Though evidence suggests that providing users with reminders to engage (also known as push notifications) can help to improve and maintain engagement, too many reminders can also have the opposite effect, resulting in disengagement [13]. Given that users of the #Here4U app will be discussing sensitive topics related to mental health and emotional well-being, combined with the associated stigma within the military community [12] and a preexisting reluctance to seek help [3,5], we made an informed decision to not implement any push notifications.

### Recommendation 14: Simple and Intuitive Interface and Interactions

The #Here4U app was designed to provide users with a sleek and simple interface. The app itself is driven by IBM’s Watson Assistant AI, which carries on text-based conversations with users to determine areas of concern and suggest potential self-care resources and other solutions. All components of the app, including tip sheets and FAQs to drive mental health education, self-help resources, and other activities, are offered within a single interface. Conversations and access to self-help resources are user led, meaning that users choose how in-depth to go with content.

### Recommendation 15: Links to Crisis Support Services

Because the #Here4U app was not intended for use as a crisis service, it was imperative to ensure that the AI had the ability to accurately recognize expressions related to self-harm and suicidality. In this case, users are reminded that the app is not equipped to assist with acute mental health crises and are directed to contact Crisis Services Canada, their primary health care provider, or local hospital.

### Recommendation 16: Experimental Trials to Establish Efficacy

To date, no evaluation work has been conducted on the efficacy of the #Here4U app, and this is therefore a major priority moving forward.

## Discussion

### Principal Findings

To our knowledge, the #Here4U app met more of Bakker and colleagues’ recommended criteria than any other available app targeting the mental health of military communities. Only 1 relevant criterion was not met: recommendation 16, to conduct experimental trials to establish efficacy of the app. While an evaluation study was out of scope for the development phase of the project, it is a recognizable gap for the #Here4U app and is therefore a major priority moving forward. Two other criteria were unmet, as they were not applicable to this project: recommendations 12 and 13, to maintain a log of past app use and to remind users to engage, respectively. Due to the sensitivity of the subject matter to be discussed within the app, combined with existing research regarding mental health–related stigma within the military community [12] and a preexisting reluctance to seek help [3,5], we made an informed decision early on to not implement any push notifications or information tracking within the #Here4U app.

### Conclusion

Although not all criteria were met by the #Here4U app, Bakker and colleagues rightly noted that it may not be possible to design mental health apps that meet all suggested criteria. It is important to note, however, that those criteria that fall within the strongest ranks of evidence as determined by Bakker and colleagues (eg, recommendations 1-8) were all met by the #Here4U app [13]. Moving forward, an experimental evaluation study should be conducted to determine the efficacy of the app and user experiences more formally. The evaluation should incorporate assessments for effectiveness of the app in connecting users with mental health education and useful self-help resources, user interface performance and usability, and general user experience. Overall, the #Here4U app may be a promising new mental health e-solution for members of the CAF community, filling many of the gaps left by traditional service delivery.

## Abbreviations

AI: artificial intelligence
CAF: Canadian Armed Forces
CBT: cognitive behavioral therapy
FAQs: frequently asked questions
PTSD: posttraumatic stress disorder

## References

[1] Daigle P (2013). On the Homefront: Assessing the Well-Being of Canada's Military Families in the New Millennium.

[2] Cramm H, Norris D, Tam-Seto L, Eichler M, Smith-Evans K (2015). Making military families in Canada a research priority. J Mil Veteran Fam Health.

[3] Zamorski MA, Rusu C, Garber BG (2014). Prevalence and correlates of mental health problems in Canadian Forces personnel who deployed in support of the mission in Afghanistan: findings from postdeployment screenings, 2009-2012. Can J Psychiatry.

[4] Mahar AL, Aiken AB, Cramm H, Whitehead M, Groome P, Kurdyak P (2018). Mental health services use trends in Canadian Veterans: a population-based retrospective cohort study in Ontario. Can J Psychiatry.

[5] Pearson C, Zamorski M, Janz T (2014). Mental Health of the Canadian Armed Forces.

[6] Zamorski MA, Boulos D (2014). The impact of the military mission in Afghanistan on mental health in the Canadian Armed Forces: a summary of research findings. Eur J Psychotraumatol.

[7] Van Til L, Sweet J, Poirier A, McKinnon K, Sudom K, Dursun S (2017). Well-Being of Canadian Regular Force Veterans, Findings from LASS 2016 survey.

[8] Cramm H, Mahar A, Tam-Seto L, Rowan-Legg A (2018). Providing care to children and youth from military families.

[9] Cramm H, Norris D, Schwartz KD, Tam-Seto L, Williams A, Mahar A (2019). Impact of Canadian Armed Forces veterans’ mental health problems on the family during the military to civilian transition. Milit Behav Health.

[10] Manser L (2018). State of Military Families in Canada: Issues Facing Regular Force Members and Their Families.

[11] Thériault F, Gabler K, Naicker K (2016). Health and Lifestyle Information Survey of Canadian Forces Personnel 2013/2014. Regular Force Report.

[12] Weeks M, Zamorski MA, Rusu C, Colman I (2017). Mental illness-related stigma in Canadian military and civilian populations: a comparison using population health survey data. Psychiatr Serv.

[13] Bakker D, Kazantzis N, Rickwood D, Rickard N (2016). Mental health smartphone apps: review and evidence-based recommendations for future developments. JMIR Ment Health.

[14] Fenn K, Byrne M (2013). The key principles of cognitive behavioural therapy. InnovAiT.

[15] Prins A, Bovin MJ, Smolenski DJ, Marx BP, Kimerling R, Jenkins-Guarnieri MA, Kaloupek DG, Schnurr PP, Kaiser AP, Leyva YE, Tiet QQ (2016). The Primary Care PTSD Screen for DSM-5 (PC-PTSD-5): development and evaluation within a veteran primary care sample. J Gen Intern Med.

[16] Kroenke K, Spitzer RL, Williams JB (2001). The PHQ-9: validity of a brief depression severity measure. J Gen Intern Med.

[17] Spitzer RL, Kroenke K, Williams JBW, Löwe B (2006). A brief measure for assessing generalized anxiety disorder: the GAD-7. Arch Intern Med.

[18] Sartorius N, Ustün TB, Lecrubier Y, Wittchen HU (1996). Depression comorbid with anxiety: results from the WHO study on psychological disorders in primary health care. Br J Psychiatry Suppl.

[19] Ginzburg K, Ein-Dor T, Solomon Z (2010). Comorbidity of posttraumatic stress disorder, anxiety and depression: a 20-year longitudinal study of war veterans. J Affect Disord.

[20] Giles DC, Newbold J (2011). Self- and other-diagnosis in user-led mental health online communities. Qual Health Res.

[21] Smith TJ, White A, Hadden L, Young AJ, Marriott BP (2014). Associations between mental health disorders and body mass index among military personnel. Am J Health Behav.

[22] Taylor MK, Hilton SM, Campbell JS, Beckerley SE, Shobe KK, Drummond SPA, Behavioral Health Needs Assessment Team (2014). Prevalence and mental health correlates of sleep disruption among military members serving in a combat zone. Mil Med.

[23] Vun E, Turner S, Sareen J, Mota N, Afifi TO, El-Gabalawy R (2018). Prevalence of comorbid chronic pain and mental health conditions in Canadian Armed Forces active personnel: analysis of a cross-sectional survey. CMAJ Open.

[24] Hourani LL, Williams TV, Kress AM (2006). Stress, mental health, and job performance among active duty military personnel: findings from the 2002 Department of Defense Health-Related Behaviors Survey. Mil Med.

[25] Meshberg-Cohen S, Kachadourian L, Black AC, Rosen MI (2017). Relationship between substance use and attitudes towards seeking professional psychological help among veterans filing PTSD claims. Addict Behav.

[26] Bray RM, Hourani LL (2007). Substance use trends among active duty military personnel: findings from the United States Department of Defense Health Related Behavior Surveys, 1980-2005. Addiction.

[27] Tyler Boden M, Kimerling R, Kulkarni M, Bonn-Miller MO, Weaver C, Trafton J (2014). Coping among military veterans with PTSD in substance use disorder treatment. J Subst Abuse Treat.

[28] (2020). Psychology Today: find a therapist.

[29] Calian (2020). Health solutions military family health portal.

[30] Adams K, Minogue V, Lucock M (2010). Nutrition and mental health recovery. Ment Health Learn Disabil Res Pract.

[31] Maller C, Townsend M, Pryor A, Brown P, St Leger L (2006). Healthy nature healthy people: 'contact with nature' as an upstream health promotion intervention for populations. Health Promot Int.

[32] Bratman GN, Hamilton JP, Daily GC (2012). The impacts of nature experience on human cognitive function and mental health. Ann N Y Acad Sci.

[33] Jacka FN, Berk M (2013). Depression, diet and exercise. Med J Aust.

